# Association between close interpersonal contact and vaccine hesitancy: Findings from a population-based survey in Canada

**DOI:** 10.3389/fpubh.2022.971333

**Published:** 2022-10-04

**Authors:** Prince A. Adu, Sarafa A. Iyaniwura, Bushra Mahmood, Dahn Jeong, Jean Damascene Makuza, Georgine Cua, Mawuena Binka, Héctor A. Velásquez García, Notice Ringa, Stanley Wong, Amanda Yu, Mike A. Irvine, Michael Otterstatter, Naveed Z. Janjua

**Affiliations:** ^1^British Columbia Centre for Disease Control, Vancouver, BC, Canada; ^2^School of Population and Public Health, University of British Columbia, Vancouver, BC, Canada; ^3^Department of Mathematics, University of British Columbia, Vancouver, BC, Canada; ^4^Department of Medicine, University of British Columbia, Vancouver, BC, Canada; ^5^Centre for Health Evaluation & Outcome Sciences, St. Paul's Hospital, Vancouver, BC, Canada

**Keywords:** interpersonal contact, COVID-19, vaccine hesitancy, transmission, Canada

## Abstract

**Background:**

Vaccine hesitancy threatens efforts to bring the coronavirus disease 2019 (COVID-19) pandemic to an end. Given that social or interpersonal contact is an important driver for COVID-19 transmission, understanding the relationship between contact rates and vaccine hesitancy may help identify appropriate targets for strategic intervention. The purpose of this study was to assess the association between interpersonal contact and COVID-19 vaccine hesitancy among a sample of unvaccinated adults in the Canadian province of British Columbia (BC).

**Methods:**

Unvaccinated individuals participating in the BC COVID-19 Population Mixing Patterns Survey (BC-Mix) were asked to indicate their level of agreement to the statement, “*I plan to get the COVID-19 vaccine*.” Multivariable multinomial logistic regression was used to assess the association between self-reported interpersonal contact and vaccine hesitancy, adjusting for age, sex, ethnicity, educational attainment, occupation, household size and region of residence. All analyses incorporated survey sampling weights based on age, sex, geography, and ethnicity.

**Results:**

Results were based on survey responses collected between March 8, 2021 and December 6, 2021, by a total of 4,515 adults aged 18 years and older. Overall, 56.7% of respondents reported that they were willing to get the COVID-19 vaccine, 27.0% were unwilling and 16.3% were undecided. We found a dose-response association between interpersonal contact and vaccine hesitancy. Compared to individuals in the lowest quartile (least contact), those in the fourth quartile (highest contact), third quartile and second quartile groups were more likely to be vaccine hesitant, with adjusted odd ratios (aORs) of 2.85 (95% CI: 2.02, 4.00), 1.91(95% CI: 1.38, 2.64), 1.78 (95% CI: 1.13, 2.82), respectively.

**Conclusion:**

Study findings show that among unvaccinated people in BC, vaccine hesitancy is greater among those who have high contact rates, and hence potentially at higher risk of acquiring and transmitting infection. This may also impact future uptake of booster doses.

## Introduction

The Coronavirus Disease 2019 (COVID-19) pandemic continues to have adverse social, economic and health impact on societies across the globe. As of August 7, 2022, over 580 million confirmed cases of (COVID-19), have been reported globally, with over 6.4 million deaths (1). The availability of approved safe and effective COVID-19 vaccines offered hope and optimism to end the COVID-19 pandemic, and a potential way to return to pre-pandemic normalcy, even though the emergence of new severe acute respiratory syndrome coronavirus 2 (SARS-CoV-2) variants presents new challenges. Current data indicates high effectiveness of the COVID-19 vaccines against infection, transmission, severe disease, and death (2–6).

However, the potential population-level reduction in transmission, morbidity, and mortality due to COVID-19 ultimately depends on high vaccine uptake which is in turn threatened by vaccine hesitancy, a complex and context specific behavior defined as “the delay in acceptance or refusal of vaccines despite availability of vaccination services” (7). In fact, due to the recent global resurgence of highly infectious vaccine-preventable diseases such as measles, the World Health Organization (WHO) named vaccine hesitancy as one of the ten greatest threats to global health in 2019 (8). Varying degrees of COVID-19 vaccine hesitancy have been reported across the world. In Bangladesh, two cross sectional studies showed vaccine hesitancy of 32.5% (9) and 27.4% (10). A recent scoping review of COVID-19 vaccine hesitancy in Africa showed vaccine acceptance ranged from 6.9 to 97.9% (11). A systematic review of COVID-19 vaccine hesitancy in the U.S revealed vaccine acceptance rate ranging from 12 to 91.4% (12).

As of July, 2022, the following COVID-19 vaccines had received authorization for use in Canada from Health Canada: Pfizer-BioNTech Cominarty, Moderna Spikevax, AstraZeneca Vaxzevria, Janssen (Johnson & Johnson), Novavax Nuvaxovid and Medicago Covifenz (13, 14). In British Columbia (BC), Pfizer-BioNTech Cominarty and Moderna Spikevax COVID-19 vaccine were the first to receive authorization for use on September 16, 2021, according to the British Columbia Centre for Disease Control (14). A study by Statistics Canada conducted in March/April 2021 indicated that 88% of Canadians aged 12 and older were willing to get vaccinated for COVID-19 when a vaccine is available to them or have already received one dose of the vaccine (15). Ogilvie et al. (16) also reported a 79.8% COVID-19 vaccine acceptance rate among the general population of British Columbia, Canada.

Some factors associated with COVID-19 vaccine hesitancy include age, gender, chronic medical condition, fears about COVID-19, income, employment status, ethnicity, location of residence, religion, marital status, educational level etc., (17). As individuals with high interpersonal or social contacts may have a higher risk of COVID-19 transmission, vaccine uptake among this population is critical to curbing the spread of COVID-19. Although COVID-19 vaccine uptake and interpersonal contact have been investigated independently in light of their association with COVID-19 transmission, we are not aware of any study that has examined the relationship between these two important factors that affect COVID-19 transmission dynamics. Hence, the purpose of this study was to evaluate the association between interpersonal contact rate and COVID-19 vaccine hesitancy.

## Methods

This study used data from the BC COVID-19 Population Mixing Patterns Survey (BC-Mix), a repeated online survey developed to assess population mixing patterns in BC during the COVID-19 pandemic. The ongoing survey was launched in September 2020 and is open to all BC residents aged 18 years or older. Anonymous links to the survey are circulated *via* advertisements placed on social media platforms (including Facebook, Instagram, YouTube and Twitter), on flyers distributed at grocery stores, community centers and places of worship, including those frequented by ethnic minority groups. Suspected duplicate responses are removed prior to analyses. Also, survey responses that do not have completion rate of at least 33%, and valid non-missing responses for the sex and age questions are excluded for weighting and further analyses. Using the 2016 Census data (18) as reference, the survey data is weighted with the following auxiliary variables: age, sex, geography, and ethnicity, using the *weighting adjustment technique* (19). As of June 2022, more than 88,000 individuals had participated in the survey. Further details about the survey development, design and domains are described in detail elsewhere (20–22). We followed the checklist for Reporting Results of Internet E-Surveys (CHERRIES) (23). The domain on vaccine hesitancy was added to the survey on March 8, 2021.

### Measures

To assess vaccine hesitancy, participants were asked whether they had received any of the approved COVID-19 vaccines. Individuals who answered that they had not yet received the COVID-19 vaccine at the time of the survey were asked to indicate their level of agreement with the statement, “*I plan to get the COVID-19 vaccine*.” Responses were rated on a five-point scale ranging from 1 to 5, with 1 being “*Strongly disagree*” and 5 being “*Strongly agree*.” For the purposes of analyses, the responses were recoded, with those who responded, “*Strongly Disagree*” or “*Disagree*” coded as “*unwilling to receive COVID-19 vaccine or vaccine hesitant*” and those who responded “*Agree*” and “*Strongly Agree*” coded as “*willing to receive a COVID-19 vaccine*.” Individuals who chose “*Neutral*” were considered “*undecided*.”

Interpersonal contact was assessed by the number of in-person, face-to-face contacts that a participant had within the past 24 h. The number of contacts was categorized by quartiles.

We assessed age, sex, ethnicity, educational attainment, occupation, household size, employment status and health region of participants (24), based on self-reported data from survey questions. The literature guided our choice of these characteristics as confounders in our assessment of the association between interpersonal contact and vaccine hesitancy.

Additional variables such as material and social deprivation index were derived using census and location data (25). Further details (including definitions and response categories) on all the survey questions relevant to this study are provided in Supplementary Table 1 of the Supplementary material.

### Analyses

Participant characteristics were summarized using weighted frequencies and percentages and are presented in Table 1. Survey methodology and weighting technique have been described elsewhere (20). Characteristics of study participants were stratified by contact rate (Table 2) and also by COVID-19 vaccine hesitancy (Table 3).

**Table 1:** Characteristics of study participants (unweighted *N* = 4,515), March 8, 2021-December 6, 2021.

		***N***	**Weighted *N***	**Weighted % (95% CI)**
**Willingness to receive vaccine**				
	Hesitant	1,028	1,561	27.0 (24.9, 29.2)
	Willing	2,903	3,273	56.7 (54.3, 59.1)
	Undecided	584	939	16.3 (14.3, 18.2)
**Sex**				
	Male	1,159	3,590	62.2 (60.1, 64.2)
	Female	3,356	2,183	37.8 (35.8, 39.9)
**Age**				
	18–34	623	1,728	29.9 (27.4, 32.4)
	35–54	1,542	2,161	37.4 (35.1, 39.8)
	55+	2,350	1,884	32.6 (30.6, 34.7)
**Ethnicity**				
	Chinese	79	392	6.8 (5.1, 8.5)
	Not a visible minority (White)	3,623	3,443	59.6 (57.1, 62.2)
	South Asian	101	586	10.1 (7.9, 12.4)
	Other ethnicities	458	955	16.5 (14.7, 18.4)
	Missing/Unknown	254	396	6.9 (5.7, 8.1)
**Educational attainment**				
	Below high school	122	181	3.1 (2.3, 4.0)
	Below bachelor	1,818	2,166	37.5 (35.2, 39.9)
	University degree	1,351	1,701	29.5 (27.2, 31.7)
	Missing/Unknown	1,224	1,725	29.9 (27.7, 32.1)
**Employment status**				
	Employed full-time (30 h or more/week)	1,100	1,708	29.6 (27.2, 31.9)
	Employed part-time (< 30 h/week)	283	392	6.8 (5.4, 8.1)
	Self-employed	352	457	7.9 (6.5, 9.3)
	Unemployed but looking for a job	136	207	3.6 (2.7, 4.4)
	Unemployed and not looking for a job	63	88	1.5 (1.0, 2.1)
	Full-time parent, homemaker	138	128	2.2 (1.7, 2.8)
	Retired	1,051	791	13.7 (12–15)
	Student/Pupil	53	159	2.7 (1.8, 3.7)
	Long-term sick or disabled	128	145	2.5 (1.8, 3.2)
	Prefer not to answer	92	163	2.8 (1.8, 3.8)
	Missing/Unknown	1,119	1,536	26.6 (24.5, 28.7)
**Occupation**				
	Essential workers	862	1,343	23.3 (21.1, 25.4)
	Non-essential workers	1,137	1,338	23.2 (21.1, 26.3)
	Do not work	941	880	15.2 (13.6, 16.9)
	Other occupations	323	465	8.0 (6.6, 9.5)
	Prefer not to answer	133	212	3.7 (2.7, 4.6)
	Missing/Unknown	1,119	1,536	26.6 (24.5, 28.7)
**Household size**				
	1	809	849	14.7 (13.2, 16.2)
	2	1,978	2,090	36.2 (34.0, 38.4)
	3	593	883	15.3 (13.4, 17.2)
	4	635	887	15.4 (13.5, 17.2)
	5	181	333	5.8 (4.4, 7.1)
	6+	292	663	11.5 (9.6, 13.3)
	Prefer not to answer	27	68	1.2 (0.5, 1.9)
**Health region**				
	Interior	753	877	15.2 (13.6, 16.8)
	Fraser	831	1,510	26.2 (23.6, 28.7)
	Vancouver Coastal	681	827	14.3 (12.7, 15.9)
	Vancouver Island	869	673	11.7 (10.4, 12.9)
	Northern	213	275	4.8 (3.8, 5.7)
	Missing/Unknown	1,168	1,611	27.9 (25.8, 30.0)
**Material deprivation**				
	1 (Privileged)	619	649	11.2 (9.9, 12.6)
	2	580	749	13.0 (11.3, 14.6)
	3	685	724	12.5 (11.0, 14.1)
	4	527	670	11.6 (10.1, 13.2)
	5 (Deprived)	491	775	13.4 (11.4, 15.5)
	Missing/Unknown	1,613	2,205	38.2 (35.9, 40.5)
**Social deprivation**				
	1 (Privileged)	451	639	11.1 (9.3, 12.8)
	2	458	544	9.4 (8.0, 10.9)
	3	693	826	14.3 (12.5, 16.1)
	4	576	709	12.3 (10.6, 13.9)
	5 (Deprived)	724	850	14.7 (13.2, 16.3)
	Missing/Unknown	1,613	2,205	38.2 (35.9, 40.5)

**Table 2:** Characteristics of study participants by interpersonal contact (unweighted, *N* = 4,515), March 8, 2021- December 6, 2021.

		**Q1 (lowest)**		**Q2**		**Q3**		**Q4 (highest)**	
		**Weighted**,	***N* % (95% CI)**	**Weighted**,	***N* % (95% CI)**	**Weighted**,	***N* % (95% CI)**	**Weighted**,	***N* % (95% CI)**
**Sex**									
	Male	1,378	38.4 (34.7, 42.1)	420	11.7 (9.4, 14.0)	754	20.9 (18.2, 23.8)	1,037	28.9 (25.7, 32.1)
	Female	875	40.0 (38.0, 42.5)	290	13.3 (11.6, 15.0)	496	22.7 (20.7, 24.7)	523	23.9 (21.8, 26.0)
**Age**									
	18–34	621	36.0 (30.8, 41.1)	214	12.4 (8.9, 15.8)	351	20.3 (16.0, 24.6)	542	31.4 (26.6, 36.2)
	35–54	792	37.0 (32.5, 40.8)	245	11.3 (8.6, 14.0)	509	23.6 (20.5, 26.7)	614	28.4 (25.0, 31.9)
	55+	839	45.0 (41.2, 47.9)	252	13.4 (11.4, 15.3)	390	20.7 (18.2, 23.2)	403	21.4 (18.7, 24.1)
**Ethnicity**									
	Chinese	190	48.4 (35.2, 61.6)	55	14.1 (5.4, 22.8)	75	19.2 (9.1, 29.4)	71	18.2 (8.7, 27.8)
	Not a visible minority (White)	1,233	35.8 (33.4, 38.2)	443	12.9 (11.2, 14.5)	834	24.2 (22.1, 26.4)	933	27.1 (24.7, 29.5)
	South Asian	341	58.3 (46.3, 70.2)	69	11.8 (3.4, 20.1)	69	11.9 (4.7, 19.0)	106	18.1 (9.7, 26.6)
	Other ethnicities	366	38.3 (32.4, 44.2)	117	12.2 (8.4, 16.0)	187	19.6 (14.7, 24.4)	286	30.0 (24.6, 35.3)
	Missing/Unknown	123	31.0 (23.3, 38.7)	27	6.8 (2.8, 10.8)	84	21.3 (13.8, 28.7)	162	40.9 (31.7, 50.1)
**Educational attainment**									
	Below high school	102	56.7 (43.2, 70.2)	32	17.5 (7.9, 27.2)	25	13.9 (6.8, 20.9)	22	11.9 (3.2, 20.6)
	Below bachelor	992	45.8 (41.8, 49.8)	278	12.9 (10.4, 15.4)	498	23.0 (19.9, 26.1)	398	18.4 (15.0, 21.7)
	University degree	822	48.3 (43.7, 53.0)	256	15.1 (11.7, 18.4)	365	21.5 (17.9, 25.0)	257	15.1 (12.1, 18.1)
	Missing/Unknown	336	19.5 (15.7, 23.3)	144	8.3 (6.0, 10.7)	361	21.0 (17.4, 24.5)	883	51.2 (46.9, 55.6)
**Employment status**									
	Employed full-time (30 h or more/week)	657	38.5 (33.6, 43.4)	226	13.3 (9.9, 16.6)	420	24.6 (20.5, 28.7)	404	23.7 (19.6, 27.7)
	Employed part-time (< 30 h/week)	176	44.9 (34.4, 55.4)	70	18.0 (9.6, 26.4)	74	19.0 (12.3, 25.6)	71	18.1 (9.4, 26.8)
	Self-employed	207	45.2 (35.7, 54.9)	73	16.1 (9.7, 22.5)	98	21.5 (15.4, 27.7)	78	17.1 (10.9, 23.3)
	Unemployed but looking for a job	128	61.9 (49.5, 74.2)	15	7.1 (2.8, 11.4)	39	19.1 (10.0, 28.2)	25	12.0 (0.6, 23.3)
	Unemployed and not looking for a job	57	64.1 (46.6, 81.6)	10	10.9 (1.3, 20.5)	14	16.4 (4.5, 28.3)	8	8.6 (0.0, 19.7)
	Full-time parent, homemaker	59	46.1 (33.8, 58.4)	16	12.3 (6.1, 18.5)	45	34.8 (23.5, 46.0)	9	6.8 (2.6, 11.0)
	Retired	473	59.8 (55.1, 64.5)	114	14.4 (11.4, 17.4)	146	18.4 (15.3, 21.6)	58	7.4 (4.8, 10.0)
	Student/Pupil	83	52.5 (34.6, 70.3)	28	17.5 (4.7, 30.3)	30	19.2 (3.8, 34.6)	17	10.8 (0.8, 20.8)
	Long-term sick or disabled	87	59.7 (46.8, 72.6)	20	13.8 (5.9, 21.6)	30	20.7 (10.2, 31.1)	9	5.9 (0.4, 11.4)
	Prefer not to answer	97	59.8 (42.9, 76.6)	12	7.4 (0.0, 16.9)	10	6.2 (1.4, 11.0)	43	26.7 (11.9, 41.4)
	Missing/Unknown	230	14.9 (11.6, 18.3)	127	8.2 (5.8, 10.7)	342	22.3 (18.4, 26.1)	838	54.5 (50.1, 59.0)
**Occupation**									
	Essential workers	516	38.4 (33.3, 43.6)	178	13.3 (9.4, 17.2)	340	25.3 (20.8, 29.9)	308	23.0 (18.5, 27.4)
	Non-essential workers	638	47.7 (42.5, 52.9)	216	16.2 (12.7, 19.6)	280	21.0 (17.3, 24.6)	203	15.2 (11.8, 18.6)
	Do not work	540	61.4 (56.0, 66.8)	118	13.4 (9.5, 17.2)	161	18.3 (14.4, 22.2)	61	7.0 (4.2, 9.8)
	Others	221	47.5 (37.8, 57.2)	52	11.3 (5.9, 16.6)	91	19.6 (13.5, 25.7)	101	21.6 (12.7, 30.6)
	Prefer not to answer	109	51.5 (37.9, 65.0)	19	9.1 (1.4, 16.9)	35	16.7 (7.2, 26.2)	48	22.7 (11.8, 33.7)
	Missing/Unknown	230	14.9 (11.6, 18.3)	127	8.2 (5.8, 10.7)	342	22.3 (18.4, 26.1)	838	54.5 (50.1, 59.0)
**Household size**									
	1	429	50.5 (45.0, 56.0)	79	9.4 (5.9, 12.8)	149	17.6 (13.7, 21.4)	192	22.6 (18.0, 27.2)
	2	986	47.2 (43.7, 50.7)	261	12.5 (10.2, 14.8)	363	17.4 (14.9, 19.8)	479	22.9 (20.0, 25.9)
	3	343	38.9 (31.9, 45.8)	122	13.8 (9.8, 17.9)	177	20.1 (15.3, 24.9)	241	27.3 (21.2, 33.3)
	4	253	28.5 (21.8, 35.1)	132	14.9 (11.0, 18.8)	280	31.5 (25.7, 37.4)	223	25.1 (19.8, 30.4)
	5	69	20.9 (9.7, 32.1)	33	10.0 (3.4, 16.5)	105	31.6 (20.6, 42.5)	125	37.6 (26.4, 48.8)
	6+	140	21.1 (13.0, 29.3)	81	12.3 (5.1, 19.4)	170	25.6 (18.4, 32.7)	272	41.0 (32.7, 49.3)
	Prefer not to answer	32	47.5 (15.2, 79.9)	1	1.9 (0.0, 5.9)	6	9.1 (0.0, 19.1)	28	41.5 (11.1, 72.0)
**Health region**									
	Interior	344	39.2 (33.9, 44.6)	128	14.6 (10.5, 18.7)	202	23.0 (18.4, 27.6)	203	23.2 (18.1, 28.2)
	Fraser	744	49.3 (43.2, 55.3)	186	12.3 (8.5, 16.1)	314	20.8 (16.4, 25.2)	266	17.6 (13.3, 21.9)
	Vancouver Coastal	390	47.1 (41.3, 53.0)	106	12.9 (9.0, 16.7)	156	18.9 (14.5, 23.3)	175	21.1 (16.3, 25.9)
	Vancouver Island	284	42.3 (36.9, 47.6)	107	15.8 (11.9, 19.8)	169	25.1 (20.4, 29.8)	113	16.8 (12.7, 20.9)
	Northern	104	37.8 (27.9, 47.7)	33	12.1 (6.3, 17.9)	55	20.2 (13.7, 26.7)	82	29.9 (19.6, 40.1)
	Missing/Unknown	387	24.0 (20.2, 27.8)	150	9.3 (6.8, 11.9)	353	21.9 (18.3, 25.6)	721	44.7 (40.4, 49.1)
**Material deprivation**									
	1 (Privileged)	292	45.0 (39.0, 51.0)	70	10.8 (7.3, 14.3)	173	26.7 (20.9, 32.4)	114	17.6 (12.8, 22.4)
	2	342	45.7 (38.9, 52.4)	124	16.5 (11.2, 21.8)	148	19.7 (15.0, 24.4)	136	18.1 (12.8, 23.4)
	3	323	44.6 (38.0, 51.2)	116	16.0 (11.8, 20.1)	150	20.7 (15.8, 25.6)	135	18.7 (13.8, 23.6)
	4	315	47.0 (39.9, 54.3)	61	9.1 (6.1, 12.1)	157	23.4 (18.1, 28.6)	137	20.5 (15.0, 26.1)
	5 (Deprived)	329	42.5 (34.0, 51.0)	135	17.4 (10.9, 23.9)	158	20.3 (14.1, 26.5)	153	19.8 (13.3, 26.3)
	Missing/Unknown	652	29.5 (26.0, 33.1)	205	9.3 (7.2, 11.4)	465	21.1 (18.0, 24.2)	884	40.1 (36.4, 43.8)
**Social deprivation**									
	1 (Privileged)	274	43.0 (34.4, 51.5)	113	17.7 (11.3, 24.1)	130	20.4 (14.4, 26.4)	121	18.9 (11.9, 25.9)
	2	207	38.1 (30.0, 46.6)	65	12.0 (7.9, 16.1)	121	22.3 (16.5, 28.1)	150	27.7 (20.3, 35.0)
	3	344	41.7 (35.0, 48.4)	121	14.6 (10.4, 18.9)	233	28.3 (22.2, 34.4)	127	15.4 (10.9, 19.9)
	4	337	47.5 (40.3, 54.7)	98	13.8 (7.9, 19.7)	148	20.9 (15.5, 26.3)	126	17.8 (12.6, 23.0)
	5 (Deprived)	438	51.6 (46.0, 57.2)	108	12.8 (9.2, 16.3)	151	17.8 (14.1, 21.5)	151	17.8 (13.7, 22.0)
	Missing/Unknown	652	29.5 (26.0, 33.1)	205	9.3 (7.2, 11.4)	465	21.1 (18.0, 24.2)	884	40.1 (36.4, 43.8)

**Table 3:** Characteristics of study participants by vaccine hesitancy (unweighted, *N* = 4,515), March 8, 2021-December 6, 2021.

		**Willing to get vaccine**		**Unwilling to get vaccine (hesitant)**		**Undecided**	
		**Weighted**,	***N* % (95% CI)**	**Weighted**,	***N* % (95% CI)**	**Weighted**,	***N* % (95% CI)**
**Interpersonal contact**							
	Q1 (lowest)	1,524	67.7 (63.8, 71.6)	385	17.1 (14.1, 20.0)	344	15.3 (12.1, 18.4)
	Q2	406	57.2 (50.3, 64.1)	182	25.6 (19.1, 32.1)	122	17.2 (11.8, 22.6)
	Q3	665	53.2 (48.3, 58.1)	367	29.4 (24.9, 33.8)	218	17.4 (13.5, 21.3)
	Q4 (highest)	677	43.4 (38.9, 47.9)	628	40.3 (35.9, 44.6)	255	16.3 (12.6, 20.1)
**Sex**							
	Male	1,798	50.1 (46.4, 53.7)	1,155	32.2 (28.9, 35.4)	637	17.7 (14.9, 20.6)
	Female	1,475	67.6 (65.3, 69.8)	406	18.6 (16.8, 20.5)	301	13.8 (12.1, 15.5)
**Age**							
	18–34	984	56.9 (51.7, 62.2)	389	22.5 (18.1, 26.9)	356	20.6 (16.2, 25.0)
	35–54	1,180	54.6 (50.6, 58.6)	646	29.9 (26.2, 33.5)	335	15.5 (12.8, 18.3)
	55+	1,109	58.9 (55.6, 62.2)	527	28.0 (25.1, 30.9)	248	13.1 (10.3, 16.0)
**Ethnicity**							
	Chinese	302	76.9 (65.2, 88.7)	52	13.2 (3.3, 23.2)	39	9.8 (1.9, 17.8)
	Not a visible minority (White)	1,960	56.9 (54.3, 59.5)	1,021	29.6 (27.2, 32.1)	463	13.4 (11.6, 15.3)
	South Asian	387	66.1 (54.3, 77.9)	66	11.3 (3.0, 19.6)	132	22.6 (12.4, 32.8)
	Other ethnicities	469	49.1 (43.1, 55.1)	283	29.6 (24.2, 35.0)	203	21.3 (16.0, 26.5)
	Missing/Unknown	155	39.2 (30.3, 48.1)	139	35.2 (26.7, 43.6)	102	25.7 (17.6, 33.7)
**Educational attainment**							
	Below high school	91	50.5 (36.3, 64.8)	53	29.6 (17.0, 42.2)	36	19.9 (6.6, 33.2)
	Below bachelor	1,140	52.6 (48.6, 56.6)	604	27.9 (24.4, 31.3)	422	19.5 (16.0, 23.0)
	University degree	1,203	70.8 (66.4, 75.1)	333	19.6 (15.7, 23.4)	165	9.7 (6.9, 12.4)
	Missing/Unknown	838	48.6 (44.2, 52.9)	571	33.1 (29.1, 37.1)	316	18.3 (14.9, 21.7)
**Employment status**							
	Employed full-time (30 h or more/week)	964	56.5 (51.5, 61.4)	454	26.6 (22.2, 31.0)	289	16.9 (12.9, 21.0)
	Employed part-time (< 30 h/week)	265	67.6 (57.9, 77.3)	53	13.5 (7.1, 20.0)	74	18.9 (10.4, 27.4)
	Self-employed	245	53.7 (44.6, 62.8)	153	33.6 (25.6, 41.5)	58	12.7 (7.1, 18.4)
	Unemployed but looking for a job	123	59.5 (47.5, 71.5)	51	24.7 (15.1, 34.3)	33	15.7 (5.8, 25.7)
	Unemployed and not looking for a job	54	60.9 (43.2, 78.6)	28	31.8 (15.7, 47.9)	6	7.3 (0.0, 15.7)
	Full-time parent, homemaker	57	44.7 (33.1, 56.4)	36	28.1 (15.9, 40.3)	35	27.2 (16.3, 38.1)
	Retired	553	69.9 (64.7, 75.1)	153	19.4 (15.2, 23.6)	85	10.7 (6.4, 15.0)
	Student/Pupil	119	75.1 (59.9, 90.4)	19	11.8 (0.0, 24.4)	21	13.1 (2.5, 23.7)
	Long-term sick or disabled	85	58.4 (45.2, 71.6)	34	23.7 (12.5, 34.9)	26	17.9 (8.3, 27.4)
	Prefer not to answer	61	37.6 (19.9, 55.4)	71	43.5 (25.7, 61.2)	31	18.9 (5.9, 31.8)
	Missing/Unknown	747	48.6 (44.1, 53.1)	508	33.1 (29.0, 37.2)	281	18.3 (14.8, 21.8)
**Occupation**							
	Essential workers	601	44.8 (39.5, 50.0)	490	36.5 (31.3, 41.7)	251	18.7 (14.5, 23.0)
	Non-essential workers	930	69.5 (64.7, 74.3)	269	20.1 (16.1, 24.1)	139	10.4 (6.8, 14.0)
	Do not work	626	71.1 (65.9, 76.4)	132	15.1 (11.8, 18.3)	122	13.8 (9.1, 18.6)
	Others	297	64.0 (55.0, 73.0)	76	16.3 (10.9, 21.6)	92	19.7 (11.4, 28.0)
	Prefer not to answer	71	33.7 (21.6, 45.9)	87	40.9 (27.0, 54.8)	54	25.4 (13.9, 36.8)
	Missing/Unknown	747	48.6 (44.1, 53.1)	508	33.1 (29.0, 37.2)	281	18.3 (14.58, 21.8)
**Household size**							
	1	473	55.7 (50.2, 61.3)	234	27.5 (22.8, 32.3)	142	16.7 (11.9, 21.6)
	2	1,351	64.6 (61.3, 68.0)	472	22.6 (19.8, 25.3)	268	12.8 (10.1, 15.5)
	3	463	52.4 (45.6, 59.3)	258	29.3 (23.3, 35.3)	161	18.3 (12.2, 24.3)
	4	513	57.8 (51.5, 64.1)	208	23.5 (18.4, 28.5)	166	18.7 (13.8, 23.7)
	5	198	59.6 (48.5, 70.6)	69	20.7 (12.9, 28.6)	66	19.7 (10.9, 28.5)
	6+	262	39.5 (30.7, 48.2)	276	41.7 (33.0, 50.4)	125	18.9 (12.5, 25.2)
	Prefer not to answer	13	19.6 (0.0, 45.1)	44	65.0 (36.2, 93.7)	10	15.4 (0.0, 32.5)
**Health region**							
	Interior	415	47.3 (41.8, 52.8)	315	35.9 (30.5, 41.3)	147	16.8 (12.3, 21.3)
	Fraser	945	62.6 (56.7, 68.4)	283	18.7 (14.2, 23.3)	282	18.7 (13.7, 23.6)
	Vancouver Coastal	536	64.8 (59.1, 70.4)	217	26.3 (21.0, 31.5)	74	9.0 (5.7, 12.2)
	Vancouver Island	475	70.5 (65.3, 75.7)	126	18.7 (14.3, 23.1)	73	10.8 (7.0, 14.6)
	Northern	92	33.5 (24.0, 43.0)	116	42.1 (32.0, 52.3)	67	24.4 (16.2, 32.6)
	Missing/Unknown	811	50.3 (46.0, 54.7)	505	31.3 (27.4, 35.2)	295	18.3 (14.9, 21.8)
**Material deprivation**							
	Q1 (Privileged)	437	67.3 (61.4, 73.2)	141	21.7 (16.5, 26.9)	71	11.0 (6.9, 15.2)
	Q2	425	56.7 (50.0, 63.4)	180	24.1 (18.2, 30.0)	144	19.2 (13.9, 24.5)
	Q3	473	65.4 (59.1, 71.6)	177	24.4 (19.3, 29.6)	74	10.2 (5.2, 15.2)
	Q4	380	56.7 (49.6, 63.8)	189	28.3 (21.9, 34.6)	101	15.0 (9.5, 20.5)
	Q5 (Deprived)	417	53.8 (45.4, 62.2)	208	26.9 (19.5, 34.3)	150	19.4 (12.7, 26.0)
	Missing/Unknown	1,141	51.7 (47.9, 55.5)	666	30.2 (26.9, 33.5)	399	18.1 (15.0, 21.2)
**Social deprivation**							
	1 (Privileged)	420	65.8 (57.7, 73.9)	111	17.4 (11.7, 23.1)	107	16.8 (9.7, 23.9)
	2	321	59.0 (50.8, 67.2)	153	28.2 (20.6, 35.8)	70	12.8 (7.3, 18.3)
	3	488	59.1 (52.5, 65.7)	218	26.4 (20.8, 32.0)	119	14.5 (9.2, 19.7)
	4	406	57.2 (49.8, 64.6)	192	27.1 (20.1, 34.2)	111	15.7 (10.0, 21.3)
	5 (Deprived)	497	58.5 (53.0, 64.0)	220	25.9 (21.1, 30.8)	132	15.6 (11.7, 19.5)
	Missing/Unknown	1,141	51.7 (47.9, 55.5)	666	30.2 (26.9, 33.5)	399	18.1 (15.0, 21.2)

We investigated the association between interpersonal contact (primary exposure) and COVID-19 vaccine hesitancy (outcome measure) while accounting for demographic and other variables using multivariable multinomial logistic regression (Table 4). In a sensitivity analysis, we repeated this analysis but for the outcome variable, we considered those who had already received the vaccine as willing to receive it (Supplementary Tables 2, 3 of the Supplementary material).

**Table 4:** Multivariable multinomial logistic regression model for association between interpersonal contact and vaccine hesitancy, March 8, 2021-December 6, 2021.

		**Undecided**	**Unwilling to get vaccine**
		**Adjusted OR (95% CI)**	**Adjusted OR (95% CI)**
**Interpersonal contact**			
	Q1	Reference	Reference
	Q2	1.41 (0.88, 2.25)	**1.78 (1.13, 2.82)**
	Q3	1.38 (0.94, 2.03)	**1.91 (1.38, 2.64)**
	Q4	1.28 (0.80, 2.04)	**2.85 (2.02, 4.00)**
**Sex**			
	Male	Reference	Reference
	Female	**0.63 (0.48, 0.83)**	**0.49 (0.40, 0.61)**
**Age**			
	18–34	Reference	Reference
	35–54	0.87 (0.59, 1.29)	**1.47 (1.04, 2.08)**
	55+	0.81 (0.53, 1.25)	**1.75 (1.24, 2.48)**
**Ethnicity**			
	Not a visible minority (White)	Reference	Reference
	Chinese	0.77 (0.31, 1.96)	0.48 (0.20, 1.13)
	South Asian	1.44 (0.73, 2.84)	**0.41 (0.20, 0.86)**
	Other	**1.72 (1.15, 2.55)**	1.22 (0.87, 1.71)
	Missing/Unknown	**2.40 (1.37, 4.19)**	1.38 (0.82, 2.34)
**Educational attainment**			
	Below high school	Reference	Reference
	Below bachelor	1.06 (0.41, 2.73)	0.74 (0.37, 1.50)
	University degree	0.43 (0.16, 1.14)	**0.46 (0.22, 0.96)**
	Missing/Unknown	0.61 (0.17, 2.12)	0.56 (0.19, 1.70)
**Occupation**			
	Essential workers	Reference	Reference
	Non-essential workers	**0.53 (0.32, 0.89)**	**0.52 (0.35, 0.75)**
	Do not work	**0.56 (0.33, 0.95)**	**0.33 (0.22, 0.49)**
	Other occupations	0.84 (0.45, 1.54)	**0.36 (0.22, 0.60)**
	Prefer not to answer	1.82 (0.82, 4.07)	1.48 (0.74, 2.94)
	Missing/Unknown	1.07 (0.37, 3.10)	0.62 (0.24, 1.65)
**Household size**			
	1	Reference	Reference
	2	**0.57 (0.36, 0.88)**	**0.63 (0.46, 0.87)**
	3	0.90 (0.52, 1.56)	1.05 (0.67, 1.64)
	4	0.80 (0.47, 1.36)	0.78 (0.51, 1.19)
	5	0.68 (0.32, 1.47)	0.58 (0.31, 1.06)
	6+	1.04 (0.55, 1.99)	**1.74 (1.06, 2.84)**
	Prefer not to answer	0.88 (0.10, 7.72)	5.74 (0.86, 38.59)
**Health region**			
	Interior	Reference	Reference
	Fraser	0.87 (0.54, 1.39)	**0.47 (0.31, 0.70)**
	Vancouver Coastal	**0.47 (0.28, 0.79)**	0.77 (0.52, 1.14)
	Vancouver Island	**0.45 (0.27, 0.73)**	**0.39 (0.26, 0.58)**
	Northern	1.96 (1.00, 3.84)	1.43 (0.80, 2.57)
	Missing/Unknown	0.90 (0.53, 1.53)	0.83 (0.54, 1.27)

“Willing to get vaccine” was the reference group in the multinomial logistic regression model. In bold are results whose confidence intervals do not include 1.

All analyses incorporated survey sampling weights that were estimated based on age, sex, geography (region), and ethnicity distribution as described elsewhere (20). All tests were two-sided significant at the 0.05 level. Analyses were performed in SAS software version 9.4 (26).

### Ethics approval

Informed consent was obtained from all participants. Ethical approval for this study was provided by the University of British Columbia Behavioral Research Ethics Board (No: H20-01785).

## Results

### Participant characteristics (overall)

Out of 15,796 respondents completing the survey between March 8 and December 6, 2021, 11,127 (70.4%) had received COVID-19 vaccine and 154 (1.0%) had missing/non-valid responses, so were ineligible for analysis, leaving 4,515 (28.6%) eligible records for analysis (i.e., people who had not yet been vaccinated and who provided valid responses to the willingness to get vaccinated question). The results presented here are based on weighted survey responses from these 4,515 responses.

The majority of participants were male (62.2%), between 35 and 54 years old (37.4%), identified as White (59.6%), had a full-time employment (29.6%), and lived with one other person in their household (36.2%). The Fraser Health region contributed the largest number of participants (26.2%). Also, 37.5% of respondents had below bachelor's education, while 29.5% had a University degree (Table 1).

Overall, 56.7% of respondents reported that they were willing to get the COVID-19 vaccine, 27.0% were unwilling (vaccine hesitant) and 16.3% were undecided about getting the COVID-19 vaccine (Table 1). However, when people who had already received the vaccine were included with those who said were willing to receive the vaccine, the proportion willing to get the vaccine was 86.5% (Supplementary Table 2 in the Supplementary material).

### Participant characteristics by interpersonal contact

Characteristics of study participants by interpersonal contact are summarized in Table 2. Whereas, 28.9% of contacts made by males were in the highest quartile, only 23.9% of contacts made by females were in the highest quartile of interpersonal contacts. Among the 18–34 years age group, 31.4% of contacts were in the highest quartile, compared to 28.4 and 21.4% in the highest quartile among the 35–54 and ≥55 years age groups, respectively. Whereas, 23.0% of contacts made by essential workers were in the highest quartile, only 15.2% of contacts made by non-essential workers were in the highest quartile of interpersonal contacts distribution.

### Participant characteristics by vaccine hesitancy

The characteristics of study participants by vaccine hesitancy are presented in Table 3. Among individuals with the least interpersonal contacts, only 17.1% were vaccine hesitant compared to 40.3% among those with the highest interpersonal contact. Whereas, 32.2% of males were vaccine hesitant, only 18.6% of females were deemed vaccine hesitant. Also, 36.5% of essential workers were vaccine hesitant compared to 20.1% of non-essential workers. Vaccine hesitancy was identified in 29.6% of Whites, 13.2% of Chinese, and 11.3% of South Asians. Individuals in large households (≥ 6 household members) were more likely to report vaccine hesitancy, compared to those in smaller households. Also, people in the most privileged quintiles (Q1) of both material and social deprivation indices were more willing to receive the COVID-19 vaccine (67.3 and 65.8 %, respectively) compared to those in the least privileged (correspondingly 53.8 and 58.5%).

### Association between interpersonal contact and vaccine hesitancy

Results from the multivariable multinomial logistic regression model assessing the association between interpersonal contact and vaccine hesitancy are shown in Table 4. In the model, we found a dose-response association between interpersonal contact and vaccine hesitancy; compared to individuals in the lowest quartile (least contact), those in the fourth quartile (reporting the highest number of contacts), third quartile and second quartile were more likely to be vaccine hesitant, adjusted odd ratios (aORs) 2.85 (95% CI: 2.02, 4.00), 1.91 (95% CI: 1.38, 2.64) and 1.78 (95% CI: 1.13, 2.82). In the sensitivity analysis where the outcome variable (willingness to vaccinate) included individuals who had already received the vaccine, we also found that compared to individuals in the lowest quartile (least contact), those in the fourth quartile (highest contact) were more likely to be vaccine hesitant, aOR =1.65 (95% CI: 1.26, 2.16) (Supplementary Table 3 of the Supplementary File).

## Discussion

To our knowledge, this is the first study to investigate the association between vaccine hesitancy and interpersonal contacts, a major risk factor for COVID-19 transmission. Overall, we found that 56.7% of our study population was willing to receive COVID-19 vaccines. This compares to the 79.8% vaccine acceptance reported in a study (16) conducted in the same province (BC), almost a year prior to our study. Although differences in the time periods in which the two studies were conducted could account for the variability in these rates, some of differences may be related to the differences in sample characteristics. Specifically, whereas our study drew a sample of BC residents from social media, the previous study sampled from research cohorts who had consented to be contacted for future research and therefore participants in that study would be more likely to be health conscious and thus less likely to be vaccine hesitant. Also, because we surveyed at a time when a larger proportion of the population had already received vaccines, the sample population remaining to receive the vaccine was more likely to be composed of people with anti-vaccination sentiments. In our sensitivity analyses, where individuals who had received the vaccine were included with those who indicated they were willing to be vaccinated, vaccine acceptance was 86.5%, slightly higher than the previous study (16).

We found that individuals with high interpersonal contact were more likely to be vaccine hesitant compared to those with low contacts. Specifically, we found a dose-response association between interpersonal contact and vaccine hesitancy; compared to individuals in the lowest quartile (least contact), those in the fourth quartile (i.e., those with the highest contact), third quartile and second quartile groups had 185, 91, and 78% increased odds of vaccine hesitancy, respectively. Consistently, in the sensitivity analyses where individuals who had already received were assumed to be willing to receive the vaccine, we also found that, compared to individuals in the lowest quartile, those in the fourth quartile of interpersonal contact had a 65% increased odds of vaccine hesitancy. These findings are concerning, given that COVID-19 transmission is driven by interpersonal contact. Therefore, vaccine hesitancy among people who are more likely to transmit the virus due to their high levels of interpersonal contacts, presents a major threat to the expected gains from current and future vaccination efforts.

The gendered patterns of vaccine hesitancy from the COVID-19 literature were also reflected in our study. Contrary to other findings (16, 27, 28) our investigation showed that the likelihood of getting a COVID-19 vaccine was higher among females than males. This discrepancy may be due to differences in the type of sample [as discussed previously in regard to (16)], and time frame [responses in the study by (27) were collected before vaccines were widely available in the U.S.]. Nonetheless, more research with population-based samples is required to elucidate this matter.

Another concerning finding was that individuals who lived in larger households (six or more people in household) were more likely to be vaccine hesitant. This is alarming given individuals in larger households are more likely to be in cramped spaces with limited ability to properly distance from one another.

Racial and ethnic disparities in vaccination have been highlighted in many studies (17). Paul et al. (29) report that individuals with ethnic minority backgrounds have higher distrustful attitudes toward vaccination. These attitudes, which may be fueled by anti-intellectualism and misinformation and lack of appropriate information in accessible language and formats (30), could lead to lower perceived risk of COVID-19 infection and severity of illness. Ongoing racism, historical contexts related to racism, such as unethical research trials on Black and Indigenous Peoples (31–33) may lead to skepticism, distrust and lower perceived benefits of COVID-19 vaccination among these populations. Vaccine hesitancy among ethnic and racial minorities is concerning given the disproportionate burden of COVID-19 outcomes among these groups (34, 35). Studies, particularly in the U.S and UK, have reported lower vaccine acceptance in racial minority populations (35–39). However, we found that whereas 29.6% of Whites reported vaccine hesitancy, 13.2% of Chinese and 11.3% of South Asians were vaccine hesitant in our analysis. These findings are consistent with a Canadian national survey where vaccine hesitancy was lower among South Asian and Chinese population compared to the White population, although higher among Black population (40). These findings reflect the heterogeneity and diverse experiences of minority groups across the world. The disproportionately higher burden of COVID-19 among racialized or minority groups in Canada particularly prior to vaccine availability (41) potentially highlighted the importance of vaccines in preventing further infections to members of this group; potentially making people more willing to accept vaccines. However, the higher burden of COVID-19 among South Asians population in UK did not affect vaccine hesitancy, highlighting differences in underlying beliefs, perceptions, and trust in the healthcare system. In addition, it has been largely recognized that vaccine hesitancy among minority groups, especially the Black community in the U.S, is fuelled by the deep-rooted and long-standing mistrust in the healthcare system and the government, driven by historical events in medical care and research (42, 43). However, the role of trust in the healthcare system and government in shaping vaccine acceptance among various groups is not very clear. Further investigations are needed to understand the differences in vaccine hesitancy and drivers of vaccine hesitancy among various ethnic groups in Canada.

It was expected that individuals considered as essential workers would be less vaccine hesitant. These are individuals in occupations deemed essential to not only the pandemic response but also maintaining essential services. Essential work includes occupations in the health sector (e.g., medical, social work, psychology), natural resources, agriculture and related production occupations, manufacturing and utilities, sales and service occupations, trades, transport and equipment operators and related occupations. In BC, these groups were prioritized in the roll-out of COVID-19 vaccination. We found that individuals in these occupations had higher odds of vaccine hesitancy and reported greater interpersonal contacts compared to those in non-essential work. Similar findings were uncovered by Ogilvie et al. where essential non-healthcare workers were found to have lower adjusted odds of intending to receive COVID-19 vaccine (16). A disconcerting facet to this is the heightened risk among this population, due to their unavoidably high contact rates with the public.

### Implication

Whereas, these are important findings which can inform strategic vaccine-acceptance messaging, it is expected that more routine systems are built to monitor vaccine hesitancy, to inform education and communication needs related to the pandemic control. The findings of this study can be used to inform public health interventions aimed at improving vaccine uptake.

Tackling misinformation about vaccination will be critical to reducing the morbidity and mortality of the disease. COVID-19 vaccine hesitancy has been associated with mistrust of vaccine benefit (29), safety concerns over vaccine development, and side effects (15). Unwillingness to receive the vaccine is also driven by misinformation or distrust of government and healthcare systems (44–47). Therefore, health communication strategies aimed at building trust between at-risk communities may be needed to address this issue (34). To make COVID-19 vaccination communication more effective, we can create targeted approaches to change behaviors and promote vaccination among vaccine hesitant individuals.

Motivational interviewing, which aims to support decision making by eliciting and strengthening a person's motivation to change their behavior based on their own arguments for change has been shown to be effective in increasing vaccine uptake (48). Additionally, medical reminders (49) and provider recommendations (48) are also effective strategies in promoting vaccinations. Among communities of color, where issues of misinformation and mistrust of the medical system appear to be a significant factor for vaccine hesitancy, a multipronged approach based on partnerships with trusted community resources such as faith-based leaders, community organizers, and community mentors can be a helpful tool in tackling low rates of vaccine uptake in these communities (50). Furthermore, it has been noted that the misinformation that drive vaccine hesitancy attitudes are propagated *via* social media (11). These same platforms could be used for such targeted campaigns, given their effective knowledge dissemination potential.

Findings from this investigation can also be useful to optimize predictive transmission models by including the impact of vaccine hesitancy on transmission risk.

### Limitations and strengths

Like all surveys, our findings are subject to social desirability bias. Although our online-based study minimizes the role of social desirability bias, it cannot be ruled out entirely as exerting some influence. In addition, although vaccine willingness or hesitancy has been highly volatile and changing depending on evolving information, our survey only captured respondent's attitudes at one period in time (March 2021 to December 2021). Further studies should examine changing trends of vaccine hesitancy. Despite these limitations, a major novelty to this study is our ability to account for each individual's contact behaviors. Furthermore, our study investigated the characteristics and factors associated with vaccine hesitancy among people who remain unvaccinated in the province, as this population may differ from the population who were ready to receive the vaccine as soon as it became available to them. Future vaccination strategies may need to be staggered, to target different aspects related to vaccination acceptance among hesitant subgroups.

## Conclusion

Despite public campaigns urging people to get vaccinated, vaccine hesitancy remains a challenge in pandemic response. We found vaccine hesitancy to be greater among individuals with higher interpersonal contacts, suggesting the need for targeted interventions to increase vaccine acceptance among this population.

## Data availability statement

The raw data supporting the conclusions of this article will be made available by the authors, without undue reservation.

## Ethics statement

The studies involving human participants were reviewed and approved by University of British Columbia Behavioral Research Ethics Board (No: H20-01785). The patients/participants provided their written informed consent to participate in this study.

## Author contributions

NJ and PA conceptualized the study. All authors reviewed and agreed on the final submission.

## Funding

This work was supported by Michael Smith Foundation for Health Research COVID-19 Research Response Fund (Award #: COV-2020-1183).

## Conflict of interest

Author NJ has advised and spoken for AbbVie, not related to this project or COVID-19. The remaining authors declare that the research was conducted in the absence of any commercial or financial relationships that could be construed as a potential conflict of interest.

## Publisher's note

All claims expressed in this article are solely those of the authors and do not necessarily represent those of their affiliated organizations, or those of the publisher, the editors and the reviewers. Any product that may be evaluated in this article, or claim that may be made by its manufacturer, is not guaranteed or endorsed by the publisher.
