# Supplementary material for: Association between close interpersonal contact and vaccine hesitancy: Findings from a population-based survey in Canada
Source: Front Public Health. 2022 Oct 4;10:971333. doi: 10.3389/fpubh.2022.971333 (PMC9577316; doi:10.3389/fpubh.2022.971333)
Supplement: Supplementary file 1 [file Data_Sheet_1.PDF]

Supplementary Material

Table S1. Relevant British Columbia COVID-19 Population Mixing Patterns (BC-Mix) survey questions

Variable	Question/definition	Original response categories	Derived categories
Interpersonal contact	Now we would like to ask you some questions about people you had in-person, face-to-face contact with yesterday. By in-person, face-to-face contact, we mean EITHER: A. An in-person two-way conversation with three or more words. OR B. Physical skin-to-skin contact (for example, a handshake, hug, kiss, or contact sports). This includes family members, friends, co-workers, people you spoke to in shops, bus drivers, strangers, etc... and people of ALL ages. Please do not count people you contacted only with things like telephone, text, or online. How many people did you have in-person contact with between 5 am yesterday and 5 am today?	0	Converted to quartiles
		1	
		2	
		3	
		4	
		5	
		6	
		7	
		8	
		9	
		10	
		11-15	
		16-20	
		21-25	
		26-30	
		31-35	
		36-40	
		41-45	
		46-50	
		51 or more	
		Prefer not to answer	Prefer not to answer
			Missing/Unknown
Willingness to vaccinate		1-Strongly Disagree	No

	I plan to get the COVID-19 vaccine	2-Disagree	
		3-Undecided	Undecided
		4-Agree	Yes
		5-Strongly Agree	
			Missing/Unknown
Age Group	What is your age?	18-24	18-34
		25-34	
		35-44	35-54
		45-54	
		55-64	55+
		65-74	
		75 or greater	
		Prefer not to answer	
Sex	What is your sex?	Male	Male
		Female	Female
		Other	
		Prefer not to answer	
Ethnicity	Do you consider yourself to be (check all that apply)	First Nations	Other ethnicity
		Métis	
		Inuit	
		White (European descent)	Not a visible minority
		Chinese	Chinese
		South Asian (e.g., East Indian, Pakistani, Sri Lankan)	South Asian
		Black (e.g., African or Caribbean)	Other ethnicity
		Filipino	Other ethnicity
		Latin American/Hispanic	Other ethnicity

		Southeast Asian (e.g., Vietnamese, Cambodian, Malaysian, Laotian)	Other ethnicity
		Arab	Other ethnicity
		West Asian (e.g., Iranian, Afghan)	Other ethnicity
		Korean	Other ethnicity
		Japanese	Other ethnicity
		( ) Other, prefer to self describe	Other ethnicity
		Prefer not to answer	Prefer not to answer
			Missing/Unknown
Educational attainment	What is the highest level of school you have completed or the highest degree you have received?	Less than high school degree	Below high school
		High school graduate (high school diploma or equivalent including GED)	Below bachelor
		Some college/university but no degree	Below bachelor
		Associate degree or diploma in college/university (2-year)	Below bachelor
		Bachelor's degree in college (4-year)	University Degree
		Master's degree	University Degree
		Doctoral Degree	University Degree
		Professional degree (e.g. JD, MD)	University Degree
		Prefer not to answer	
			Missing/Unknown
Employment Status	What is your current employment status?	Employed full-time (30 hours or more/week)	Employed full-time (30 hours or more/week)
		Employed part-time (less than 30 hours/week)	Employed part-time (less than 30 hours/week)
		Self-employed	Self-employed

		Unemployed but looking for a job	Unemployed but looking for a job
		Unemployed and not looking for a job	Unemployed and not looking for a job
		Full-time parent, homemaker	Full-time parent, homemaker
		Retired	Retired
		Student/Pupil	Student/Pupil
		Long-term sick or disabled	Long-term sick or disabled
		Prefer not to answer	Prefer not to answer
			Missing/Unknown
Occupation	As of March 2020, what occupation or industry have you most often worked in?	I do not work	Do not work
		Business, finance and administration occupations	Non-essential workers
		Health occupations (e.g., medical, social work, psychology)	Essential workers
		Management occupations	Non-essential workers
		Natural and applied sciences and related occupations	Non-essential workers
		Natural resources, agriculture, and related production occupations	Essential workers
		Occupations in art, culture, recreation, and sport	Non-essential workers
		Occupations in education, law and social, community and government services	Non-essential workers
		Occupations in manufacturing and utilities	Essential workers
		Sales and service occupations	Essential workers

		Trades, transport and equipment operators and related occupations	Essential workers
		( ) Other (prefer to self describe)	Others
		Prefer not to answer	Prefer not to answer
			Missing/Unknown
Household size	This is a derived variable by adding the responses to these two questions:<br><br>1. How many adults live in your household? If you live alone, choose 1. If you live with one person, choose 2, etc. By household, we mean anyone living at the same address as you, that you share a kitchen with.<br><br>2. How many children (under 18 years) live in your household? By household, we mean anyone living at the same address as you, that you share a kitchen with.	1	1
		2	2
		3	3
		4	4
		5	5
		6	6
		7	7
		8	8
		9	9
		10	10
		11	11
		12	12
		Prefer not to answer	Prefer not to answer
Health region	This is a derived variable using respondents' postal code.	Postal code	Interior Health
			Fraser Health
			Vancouver Coastal Health
			Vancouver Island Health
			Northern Health

			Missing/Unknown
Material deprivation	This is a derived variable using respondents postal code and Census data. Material deprivation is measured using the Quebec material index	Postal code	1 (Privileged)
			2
			3
			4
			5 (Deprived)
			Missing/Unknown
Social deprivation	This is a derived variable using respondents postal code and Census data. Social deprivation is measured using the Quebec social index.	Postal code	1 (Privileged)
			2
			3
			4
			5 (Deprived)
			Missing/Unknown

Table S2. Willingness to get a COVID-19 vaccine, (N=15,642)

		Frequency	Weighted freq.	Weighted %	95% CI
Willingness to receive COVID-19 vaccine	No	1,028	1,561	8.4	(7.7, 9.2)
	Yes*	14,030	15,985	86.5	(85.5, 87.4)
	Undecided	584	939	5.1	(4.4, 5.7)

* Includes those who have received COVID-19 vaccine

Table S3. Multivariable multinomial logistic regression model for association between interpersonal contact and vaccine hesitancy, March 8, 2021-December 6, 2021

		Undecided	Unwilling to vaccinate
		Adjusted OR (95% CI)	Adjusted OR (95% CI)
Interpersonal contact			
	Q1	Reference	Reference
	Q2	0.98 (0.64, 1.49)	1.30 (0.89, 1.92)
	Q3	0.84 (0.60, 1.18)	1.22 (0.93, 1.60)
	Q4	0.72 (0.47, 1.09)	**1.65 (1.26, 2.16)**
Sex			
	Male	Reference	Reference
	Female	**0.58 (0.46, 0.75)**	**0.48 (0.41, 0.58)**
Age			
	18-34	Reference	Reference
	35-54	0.72 (0.51, 1.01)	1.25 (0.93, 1.67)
	55+	**0.46 (0.31, 0.69)**	1.06 (0.80, 1.41)
Ethnicity			
	Not a visible minority (White)	Reference	Reference
	Chinese	0.59 (0.25, 1.41)	**0.34 (0.15, 0.77)**
	South Asian	1.33 (0.72, 2.43)	**0.34 (0.16, 0.70)**
	Other	**1.56 (1.10, 2.22)**	1.03 (0.79, 1.36)
	Missing/Unknown	**2.62 (1.66, 4.14)**	1.48 (0.99, 2.22)
Educational attainment			
	Below high school	Reference	Reference
	Below bachelor	1.11 (0.46, 2.68)	0.81 (0.47, 1.41)
	University degree	0.48 (0.19, 1.19)	**0.51 (0.28, 0.93)**
	Missing/Unknown	0.85 (0.28, 2.61)	0.71 (0.31, 1.63)
Occupation			
	Essential workers	Reference	Reference

	Non-essential workers	0.65 (0.41, 1.03)	**0.59 (0.43, 0.81)**
	Do not work	0.76 (0.46, 1.25)	**0.39 (0.28, 0.54)**
	Other occupations	1.07 (0.60, 1.91)	**0.48 (0.32, 0.71)**
	Prefer not to answer	1.60 (0.81, 3.16)	1.23 (0.70, 2.17)
	Missing/Unknown	0.89 (0.35, 2.29)	0.60 (0.29, 1.25)
Household size			
	1	Reference	Reference
	2	0.72 (0.48, 1.09)	**0.76 (0.59, 0.98)**
	3	1.07 (0.64, 1.79)	1.18 (0.84, 1.67)
	4	1.08 (0.68, 1.71)	0.94 (0.67, 1.32)
	5	1.18 (0.59, 2.12)	0.96 (0.60, 1.56)
	6+	1.63 (0.93, 2.85)	**2.71 (1.83, 4.03)**
	Prefer not to answer	0.76 (0.17, 3.43)	**4.51 (1.50, 13.54)**
Health region			
	Interior	Reference	Reference
	Fraser	0.75 (0.49, 1.16)	**0.44 (0.32, 0.62)**
	Vancouver Coastal	**0.38 (0.23, 0.63)**	**0.59 (0.42, 0.83)**
	Vancouver Island	**0.54 (0.34, 0.86)**	**0.47 (0.33, 0.66)**
	Northern	1.41 (0.83, 2.40)	1.05 (0.69, 1.60)
	Missing/Unknown	0.81 (0.49, 1.34)	0.72 (0.50, 1.03)

Note: "Willing to vaccinate" as the reference group in the multinomial logistic regression model includes those who have received COVID-19 vaccine
